# Is There any Relationship Between Myocardial Repolarization Parameters and the Frequency of Ventricular Premature Contractions?

**DOI:** 10.5935/abc.20180079

**Published:** 2018-07

**Authors:** Kayihan Karaman, Metin Karayakali, Arif Arisoy, Ilker Akar, Mustafa Ozturk, Ahmet Yanik, Samet Yilmaz, Atac Celik

**Affiliations:** 1 Gaziosmanpasa University Faculty of Medicine, Department of Cardiology, Tokat - Turkey; 2 Gaziosmanpasa University Faculty of Medicine, Department of Cardiovascular Surgery, Tokat - Turkey; 3 Erzurum Territorial Training and Research Hospital, Cardiology Clinic, Erzurum - Turkey; 4 Samsun Training and Research Hospital, Cardiology Clinic, Samsun - Turkey

**Keywords:** Ventricular Premature Complexes, Arrhythmias, Cardiac, Electrocardiography / methods, Cardiovascular Diseases, Obesity, Ventricular Dysfunction, Left

## Abstract

**Background:**

Ventricular premature contractions (VPCs) may trigger lethal ventricular arrhythmias in patients with structural heart disease. However, this role of VPCs in healthy people remains controversial once that not enough clinical trials are available. Recently, some myocardial repolarization markers, such as Tp-e interval, Tp-e/QT, and Tp-e/QTc ratios, have been reported to be useful for predicting lethal ventricular arrhythmias in various clinical disorders without structural heart disease.

**Objective:**

In this study, we aimed to investigate the relation between VPC frequent and myocardial repolarization markers in individuals without structural heart disease.

**Methods:**

This study included 100 patients who had complaints of dizziness and palpitations. Twelve-lead electrocardiography and 24-hour ambulatory Holter recordings were obtained from all patients. VPC burden was calculated as the total number of VPCs divided by the number of all QRS complexes in the total recording time. P-values < 0.05 were considered significant.

**Results:**

Tp-e interval and Tp-e/QTc ratio were significantly higher in patients with higher VPC burden than in patients with lower VPC burden, and a positive correlation was found between these markers and VPC burden. Tp-e (β = 1.318, p = 0.043) and Tp-e/QTc (β = -405.136, p = 0.024) in the lead V5 were identified as independent predictors of increased VPC burden.

**Conclusions:**

Tp-e interval and Tp-e/QTc ratio increased in patients with high VPC number. Our study showed that VPCs may have a negative effect on myocardial repolarization. This interaction may lead to an increased risk of malignant arrhythmias.

## Introduction

Ventricular premature contractions (VPCs) are commonly seen in the electrocardiography (ECG) of patients with hypertension, obesity, and structural heart disease. Some studies reported VPCs to occur in about 4% of the general population.^1,2^ As some patients may be asymptomatic, many patients suffer from VPC-related symptoms, such as palpitation, dizziness, dyspnea, and chest pain. In addition to these symptoms, frequent VPCs may cause more serious disorders. Recent studies on adults with frequent VPCs (> 20,000/24 h) have reported left ventricular dilation and/or dysfunction,^3,4^ diastolic dysfunction,^5^ and malignant ventricular arrhythmias in patients with structural heart disease.^6^ However, whether frequent VPCs are associated with malignant arrhythmias in individuals without structural heart disease remains uncertain.

T wave is commonly used in assessing myocardial repolarization. Increased transmural dispersion of myocardial repolarization in a normal heart is associated with their tendency toward cardiac arrhythmias. Recently, some myocardial repolarization markers, such as QT interval (QT), corrected QT (QTc), QT dispersion (QTd), Tp-e interval (Tp-e), and Tp-e/QT ratio have been found to be useful in predicting life-threatening cardiac arrhythmias in several clinical disorders without structural heart disease. Some studies showed that increased Tp-e, Tp-e/QT, and Tp-e/QTc were related to the elevated risk of occurrence of malignant ventricular arrhythmias.^7,8^ In this study, we investigated the relation between VPC burden and myocardial repolarization by using some ECG markers in individuals without structural heart disease.

## Methods

### Study population

One hundred patients with at least 1 VPC in the 12-lead ECG with diagnosis of dizziness, syncope, and palpitation without structural heart disease admitted to the Cardiology Department of our university hospital, between July 2016 and March 2017, were enrolled for this cross-sectional study. Twenty-four-hour ambulatory Holter recordings were obtained from all patients. VPC burden was calculated as the total number of VPCs divided by the number of all QRS complexes in the total recording time. A frequency of < 1% VPCs/24 h was denoted as "rare-group 1 (n = 32)", 1-5% VPCs/24 h was denoted as "occasional-group 2 (n = 36)", and > 5% VPCs/24 h was denoted as "frequent-group 3 (n = 32)". The exclusion criteria for all groups were non-reliable T waves on the ECG, atrial fibrillation, bundle branch block, moderate or severe valvular heart diseases, thyroid disorders, cardiomyopathies, congenital heart diseases, malignancy, pulmonary hypertension, electrolyte disturbances, acute coronary syndromes, heart failure, history of myocardial infarction, history of coronary artery bypass grafting, implanted permanent pacemaker, and LV segmental motion defect in the echocardiographic exam. The local ethics committee approval and informed consent from all patients were obtained.

### Electrocardiography and Holter Recordings

Twelve-lead ECGs were obtained at rest at 10 mm/mV amplitude and 25 mm/sec (Cardiofax V; Nihon Kohden Corp., Tokyo, Japan) rate, with the patient in the supine position. All ECGs were transferred to a computer through a scanner and then used for × 300% magnification using the Paint software. Holter recordings were performed by using Lifecard CF recorders (Del-Mar Reynolds). Patients were warned not to smoke and not to consume coffee and/or alcohol during the Holter recording. Measurements were performed on the computer by two cardiologists who were blinded to the clinical data of each patient. Ventricular tachycardia (VT) was defined as the line-up of at least three or more consecutive VPCs. The ventricular couplet (Vc) was defined as a sequential ordering of two VPCs.

RR interval, QRS duration, QT, and QTd were measured in all derivations. QT was defined as the time from the start of the QRS to the point at which the T wave returns to the isoelectric line. The average value of at least two readings was calculated for each lead. QTc was calculated by using Bazett's formula:^9^ QTc = QT/√R - R interval. QTd was defined as the difference between the longest and the shortest QT interval of the 12leads. Subjects with U waves in their ECGs were excluded from the study. In the measurement of Tp-e interval, the tail and tangent methods can be used, but the former is a better predictor of mortality than the latter.^10^ Thus, the tail method was used in this study. The tail method was defined as the interval from the peak to the end of the T wave to the point where the wave reached the isoelectric line.^9^ Measurement of the Tp-e interval was obtained from leads V2 and V5, which were corrected for heart rate (cTp-e).^11^ The Tp-e/QT and Tp-e/QTc ratios were calculated from these measurements.

### Echocardiographic examination

All echocardiography examinations (General Electric Vivid S5, Milwaukee, WI, USA) were performed by an experienced cardiologist in all subjects using a 2.5-3.5 MHz transducer in the left decubitus position. Two-dimensional and pulsed Doppler measurements were obtained using the criteria of the American Society of Echocardiography and the European Association of Cardiovascular Imaging.^12^ Left ventricular ejection fraction (LVEF) was assessed using Simpson's method.

### Statistical analysis

All tests were performed by using PASW Statistics (SPSS 18.0 for Windows, Inc., Chicago, IL, USA). Shapiro-Wilk test was used to test for normal distribution. Continuous variables were described as the mean (± standard deviation), and categorical variables were described as frequency (percentage). All continuous parameters were compared among groups by using one-way ANOVA. The post hoc Tukey's test was used for significant intergroup differences. Categorical factors were compared among groups using the χ^2^ test for independence. Correlations between two variables were performed by Pearson's correlation. Multiple linear regression analysis was used to evaluate the association between an increased VPC burden and independent variables that differed significantly in Pearson's correlation analyses (p < 0.1). A multivariate logistic regression analysis was performed to demonstrate the effect of presence of CAD on ECG parameters. P-values < 0.05 were considered significant.

## Results

The baseline demographics and laboratory characteristics of the three groups are summarized in Table 1. No significant difference was found among the three groups in terms of any baseline demographic or laboratory characteristic. Some baseline and ambulatory ECG parameters among the groups are shown in Table 2.

**Table 1:** Baseline characteristics, laboratory and echocardiographic parameters of the study population

Variables	Group 1 (n = 32)	Group 2 (n = 36)	Group 3 (n = 32)	p*
Age, years	49.60 ± 16.50	51.40 ± 17.00	52.10 ± 12.90	0.805
Female sex, n (%)	16.00 (50.00)	19.00 (52.80)	14.00 (43.8)	0.752
Body mass index, kg/m^2^	24.10 ± 2.50	23.60 ± 3.60	23.40 ± 4.40	0.657
Hypertension, n (%)	8.00 (25.00)	12.00 (33.30)	10.00 (31.3)	0.743
Diabetes Mellitus, n (%)	1.00 (3.10)	4.00 (11.10)	5.00 (15.6)	0.240
Coronary Artery Disease, n (%)	7.00 (21.90)	10.00 (27.80)	11.00 (34.4)	0.538
Smoking, n (%)	6.00 (18.80)	5.00 (13.90)	7.00 (21.9)	0.687
Systolic Blood Pressure (mmHg)	125.40 ± 15.40	125.10 ±14.30	122.80 ±14.00	0.737
Diastolic Blood Pressure (mmHg)	78.70 ± 7.50	77.50 ± 8.10	76.70 ± 8.90	0.638
Left Ventricle Ejection Fraction, (%)	62.80 ± 3.70	61.30 ± 4.20	60.90 ± 4.70	0.167
Interventricular Septum, (mm)	9.80 ± 0.70	10.20 ± 0.80	10.00 ± 0.80	0.460
Creatinine, mg/dl	0.82 ± 0.22	0.85 ± 0.22	0.83 ± 0.21	0.816
Neutrophil to Lymphocyte Ratio	1.90 ± 0.57	2.36 ± 1.05	2.26 ± 1.67	0.267
Hemoglobin, gr/dl	14.60 ± 1.60	14.00 ± 1.40	14.20 ± 1.80	0.345
β-blockers, n (%)	15.00 (46.90)	16.00 (44.40)	11.00 (34.4)	0.559
Angiotensin-converting Enzyme Inhibitors, n (%)	8.00 (25.00)	9.00 (25.00)	6.00 (18.8)	0.787
Angiotensin Receptor Blockers, n (%)	4.00 (12.50)	5.00 (13.90)	4.00 (12.5)	0.981
Number of patients with Vc, n (%)	9.00 (28.10)	21.00 (58.30)	21.00 (65.6)	0.006
Number of patients with VT, n (%)	3.00 (9.40)	11.00 (30.60)	12.00 (37.5)	0.028

Vc: ventricular couplet; VT: ventricular tachycardia. Data are presented as mean ± SD, or n (%). Statistically significant p values shown in bold.

* ANOVA and χ^2^ tests were performed to study differences among the three groups.

**Table 2:** Baseline and ambulatory Holter electrocardiography parameters of the study population

Variables	Group 1 (n = 32)	Group 2 (n = 36)	Group 3 (n = 32)	p values (groups)*		
				1 vs 2	1 vs 3	2 vs 3
Maximum Heart Rate (beats/min)	123.60 ± 17.10	120.40 ± 20.10	116.80 ± 13.20	0.720	0.259	0.671
Minimum Heart Rate (beats/min)	58.90 ± 7.40	54.90 ± 8.60	57.10 ± 7.30	0.097	0.638	0.481
Average Heart Rate (beats/min)	73.40 ± 13.40	72.40 ± 14.60	73.90 ± 12.00	0.940	0.980	0.855
Number of VPCs (median/24 h)	543.00 ± 288.00	2779 ± 1041	8358 ± 2911	< 0.001	< 0.001	< 0.001
Number of VPCs (median/h)	22.80 ± 12.40	117.50 ± 46.30	358.00 ± 125.20	< 0.001	< 0.001	< 0.001
Percent of VPC number (24 h)	0.50 ± 0.23	2.76 ± 1.03	7.90 ± 2.72	< 0.001	< 0.001	< 0.001
**Lead V2**						
QT (ms)	358.00 ± 22.80	378.10 ± 35.50	387.00 ± 25.30	0.013	< 0.001	0.419
QTc (ms)	414.30 ± 32.20	410.50 ± 27.00	427.30 ± 33.80	0.867	0.222	0.071
Tp-e (ms)	94.30 ± 9.40	100.50 ± 9.70	106.50 ± 7.90	0.016	< 0.001	0.023
cTp-e (ms)	108.60 ± 14.80	110.00 ± 16.30	117.70 ± 11.50	0.923	0.038	0.079
Tp-e/QT	0.26 ± 0.02	0.27 ± 0.03	0.28 ± 0.02	0.854	0.239	0.493
Tp-e/QTc	0.23 ± 0.02	0.24 ± 0.03	0.25 ± 0.03	0.007	0.001	0.689
**Lead V5**						
QT (ms)	363.70 ± 26.20	380.50 ± 41.50	389.30 ± 20.50	0.075	0.004	0.485
QTc (ms)	421.00 ± 37.00	413.00 ± 29.30	429.70 ± 29.10	0.554	0.524	0.084
Tp-e (ms)	91.30 ± 9.20	94.00 ± 12.20	101.10 ± 8.80	0.519	0.001	0.015
cTp-e (ms)	106.50 ± 15.10	102.3 ± 13.9	112.0 ± 14.0	0.453	0.280	0.018
Tp-e/QT	0.25 ± 0.02	0.25 ± 0.03	0.26 ± 0.03	0.895	0.372	0.163
Tp-e/QTc	0.22 ± 0.02	0.23 ± 0.03	0.24 ± 0.03	0.244	0.021	0.465
QTd (ms)	23.30 ± 6.40	26.3 ± 13.1	34.3 ± 13.4	0.537	0.001	0.015

QTc: corrected QT; QTd: QT dispersion; Tp-e: T wave peak-to-end interval; cTp-e: corrected Tp-e; ms: millisecond; VPC: ventricular premature contraction; Data are presented as mean ± SD. Statistically significant p values shown in bold;

* ANOVA test was performed to study differences among the three groups. The post hoc Tukey’s test was performed after ANOVA to study between groups differences for group 1 vs. group 2, group 1 vs. group 3 and group 2 vc. group 3.

According to the comparison of the ECG parameters among the three groups in lead V2, QT interval was significantly longer in groups 2 and 3 than in group 1. Tp-e interval in group 3 was significantly longer than those in groups 1 and 2. The Tp-e/QTc ratio significantly increased in groups 2 and 3 in comparison with group 1. When the groups were compared, no significant difference was found in QTc interval and Tp-e/QT ratio (Table 2).

In the comparison of ECG parameters among the three groups in lead V5, QT interval was significantly longer in group 3 than in group 1. Tp-e interval was significantly longer in group 3 than in groups 1 and 2. Tp-e/QTc ratio was significantly increased in the group 3 when compared to the group1. When the groups were compared, no significant difference was found in QTc interval and Tp-e/QT ratio (Table 2).

A total of 28 patients had coronary artery disease (CAD) (7, 10, and 11 patients in groups 1, 2, and 3, respectively). Non-critical lesions that did not cause significant narrowing were evident in the angiographic reports. The presence of CAD was greater in group 3 than in groups 1 and 2, but statistical significance was not observed (p = 0.538). In the multivariate logistic regression analysis, CAD had no effect on ECG parameters. Vc was observed in 51 patients (9, 21, and 21 patients in groups 1, 2, and 3, respectively) and VT in 26 patients (3, 11, and 12 patients in groups 1, 2, and 3, respectively). The QTd duration of group 3 was significantly longer than those in groups 1 and 2 (p = 0.001, p = 0.015, respectively).

**Figure 1:**
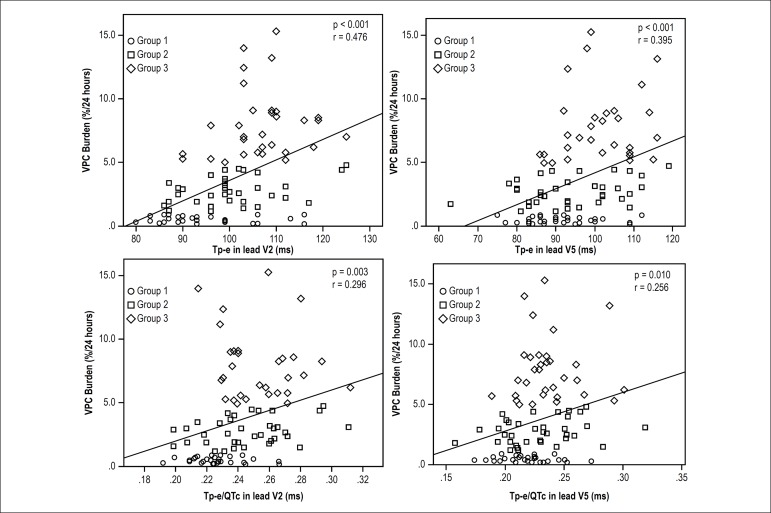
Scatter analysis of the correlation between the Tp-e interval and Tp-e/QTc ratio (in the leads V2 and V5) and the VPC burden. ms: millisecond; QTc: corrected QT; Tp-e: T wave peak-to-end interval; VPC: ventricular premature contraction.

According to Pearson's correlation test, positive correlations were observed between VPC burden and Tp-e (in leads V2 and V5) and Tp-e/QTc (in leads V2 and V5) (r = 0.476, p < 0.001; r = 0.395, p = < 0.001and r = 0.296, p = 0.003; r = 0.256, p = 0.010, respectively) (Table 3, Figure 1). Table 3 shows the results of the multiple linear regression analyses performed to identify the ECG parameters affecting VPC burden. Thus, Tp-e interval (β = 1.318, p = 0.043) and Tp-e/QTc ratio (β = -405.136, p = 0.024) in lead V5 were independent predictors of VPC burden.

**Table 3:** Relationship between ventricular premature contractions (VPC) burden and clinical and electrocardiographic parameters

Variables	VPC burden			
	Pearson correlation coefficient	p	Beta regression coefficient	p
Age	-0.026	0.797	-	-
Female sex	0.089	0.380	-	-
CAD	0.065	0.520	-	-
QTd	0.256	0.010	0.035	0.190
**Lead V2**				
QT	0.362	< 0.001	0.067	0.749
QTc	0.243	0.015	0.148	0.382
Tp-e	0.476	< 0.001	-0.665	0.260
Tp-e/QT	0.171	0.088	-48.643	0.734
Tp-e/QTc	0.296	0.003	-366.464	0.059
**Lead V5**				
QT	0.292	0.003	-0.151	0.449
QTc	0.173	0.085	-0.154	0,309
Tp-e	0.395	< 0.001	1.318	0.043
Tp-e/QT	0.185	0.066	-100.943	0.585
Tp-e/QTc	0.256	0.010	-405.136	0.024

QTc: corrected QT; QTd: QT dispersion; Tp-e: T wave peak-to-end interval; VPC: ventricular premature contraction. Pearson’s correlation and linear regression analyses.

## Discussion

In this study, we demonstrated that Tp-e interval and Tp-e/QTc ratios were significantly higher in patients with higher VPC burden than in those with lesser VPC burden and that a positive correlation was observed between these markers and VPC frequency. However, we did not find a relationship between Tp-e/QT ratio and VPC burden. Tp-e interval and Tp-e/QTc ratio in lead V5 were identified as independent predictors of increased VPC burden. The prolongation of the duration of myocardial repolarization in patients with increased VPC burden is important because this condition may be related to the increased risk of life-threatening arrhythmia. According to our results, myocardial repolarization parameters deteriorated with increasing VPC frequency. Therefore, we concluded that both VPC frequency and stage of myocardial repolarization were affected by similar causes.

Idiopathic VPCs, generally regarded as a benign condition in healthy individuals without structural heart disease, are formed by the spread of an early stimulus originating from an ectopic focus. VPCs may cause serious complications, such as angina, syncope, or heart failure, when the ectopic beat number increases.^13-16^ Although VPCs are known to be benign in individuals with a structurally normal heart, they have been shown to cause malignant arrhythmias in some cases. However, the clinical significance of VPC frequency in these individuals remains unclear once adequate human studies have not been performed.^17^ Tilz et al.^18^ found that ventricular fibrillation (VF) was triggered by VPCs after an implantable cardioverter-defibrillator was used on a 29-year-old patient, who was resuscitated following sudden cardiac death. All examinations, including echocardiography, angiography, ajmaline test, and myocardial biopsy, were normal. At the same time, some cases demonstrated that polymorphic VT and idiopathic VF were induced because specific VPCs with short coupling intervals could promote intracellular calcium overload.^19,20^ In a study examining the records of 21 patients who experienced cardiac arrest during ambulatory ECG recording, heart rate and VPC frequency increased before the onset of VF.^21^ Savelieva et al.^22^ found significant QT turbulence after VPC in individuals with a structurally healthy heart. Although these data provide information on the cause of malignant arrhythmias for VPC, they do not provide enough information about the importance of VPC frequency.

Several mechanisms have been proposed to explain the relationship between VPC and life-threatening arrhythmias. VPC may play a key role in the initiation of malignant cardiac arrhythmias. Various factors such as increased sympathetic tonus, altered hemodynamic status, or electrolyte imbalances (e.g., the hypokalemia and hypercalcemia), which all disrupt the stability of the myocardium, may cause a transition from VPC to malignant arrhythmia.^17^ Increased sympathetic tonus due to anxiety or physiological stress may cause the release of catecholamines such as adrenaline. This condition causes the flow of calcium from an extracellular space into the myocyte cells by increasing the production of cyclic AMP (cAMP). The contraction force of the myocytes increases, and the myocyte is depolarized rapidly. For this reason, myocytes become more sensitive than normal and may depolarize spontaneously without sino-atrial node depolarization. Therefore, VPC formation and frequency may increase.^23,24^ Armaganijan et al.^25^ reported the relationship of sympathetic activation with patients with ventricular arrhythmias and suggested the effectiveness of renal sympathetic denervation by catheter to reduce arrhythmic burden.

Another factor that increases the frequency of VPC is excessive caffeine consumption. Caffeine, a phosphodiesterase inhibitor, is also a central stimulant that can enhance sympathetic activity. It can increase intracellular calcium concentration by inhibiting the enzyme that catalyzes the breakdown of cAMP. Animal studies showed that caffeine administration at high doses could induce and increase the frequency of VPCs.^26,27^

Prolongation in the dispersion of myocardial repolarization predisposes the malignant ventricular arrhythmia and has prognostic importance in terms of sudden cardiac death (SCD). Prolongation of QT and QTd durations may be associated with polymorphic ventricular tachycardia, Torsades de pointes, and SCD.^28,29^ Recently, some myocardial repolarization markers, such as Tp-e interval, Tp-e/QT, and Tp-e/QTc ratios, have been reported to be useful in predicting lethal ventricular arrhythmias in various clinical disorders without structural heart disease.^7,30,31^ Tp-e interval is considered a new marker of increased risk of SCD. Yamaguchi et al.^32^ showed that Tp-e interval is more significant than QTd or QTc in predicting Torsade de Pointes in patients with acquired long QT syndrome. At the same time, an increase in Tp-e interval and Tp-e/QT ratios was shown to be associated with Brugada syndrome.^8^ Tp-e/QT and Tp-e/QTc ratios were found to be relatively more constant than other markers because they were not affected by changes in heart rate and body weight.^9^

Although we found an increase in Tp-e interval and Tp-e/QTc ratios as VPC frequency increased, the slight increase observed in Tp-e/QT ratio was not statistically significant. Yayla et al.^33^ assessed the myocardial repolarization parameters before and after RFA in patients with a VPC burden of more than 5% on a 24 h Holter recording. After the successful procedure, Tp-e interval, Tp-e/QT ratio, and Tp-e/QTc ratio significantly decreased more than before RFA (all p < 0.001). In accordance with this data, the higher detection of Tp-e interval in patients with increased VPC frequency suggests that the risk of malignant arrhythmias might be higher in these patients. In our study, malignant arrhythmias, such as Vc and VT, were seen more in group 3 patients, thus supporting our predictions. This important link can be used to closely follow up on and manage their treatment of patients with increased VPC frequency.

### Study limitations

Our study has several major limitations. First, our study was single centered and included a small number of patients. Therefore, statistical power was limited. The results should be verified in a larger prospective cohort study. Second, because we did not have other ambulatory Holter measures, such as heart rate variability and heart rate turbulence, we could not exclude the effect of these measurements on the VPC frequency. Third, we did not have data on cardiac event rates for this study because we could not follow up on the patients prospectively for future arrhythmic events. Fourth, we aimed to record a relatively young patient profile to exclude occult CAD in our study. However, we abandoned this goal because of the limited number of patients. Further comprehensive studies should be conducted with a larger number of patients and a longer follow-up time to increase the consistency of our results.

## Conclusions

In conclusion, Tp-e interval and Tp-e/QTc ratios increased in patients with high VPC number. Our study showed that VPCs could have a negative effect on myocardial repolarization. This interaction could lead to an increased risk of malignant arrhythmias.

## References

[1] Kennedy HL, Whitlock JA, Sprague MK, Kennedy LJ, Buckingham TA, Goldberg RJ (1985). Long-term follow-up of asymptomatic healthy subjects with
frequent and complex ventricular ectopy. N Engl J Med.

[2] Cheriyath P, He F, Peters I, Li X, Alagona Jr P, Wu C (2011). Relation of atrial and/or ventricular premature complexes on a
two-minute rhythm strip to the risk of sudden cardiac death: the
Atherosclerosis Risk in Communities [ARIC] study. Am J Cardiol.

[3] Bogun F, Crawford T, Reich S, Koelling TM, Armstrong W, Good E (2007). Radiofrequency ablation of frequent, idiopathic premature
ventricular complexes: comparison with a control group without
intervention. Heart Rhythm.

[4] Duffee DF, Shen WK, Smith HC (1998). Suppression of frequent premature ventricular contractions and
improvement of left ventricular function in patients with presumed
idiopathic dilated cardiomyopathy. Mayo Clin Proc.

[5] Topaloglu S, Aras D, Cagli K, Yildiz A, Cagirci G, Cay S (2007). Evaluation of left ventricular diastolic functions in patients
with frequent premature ventricular contractions from right ventricular
outflow tract. Heart Vessels.

[6] Moss AJ, Akiyama T (1974). Prognostic significance of ventricular premature
beats. Cardiovasc Clin.

[7] Karaman K, Altunkas F, Çetin M, Karayakali M, Arisoy A, Akar I (2015). New markers for ventricular repolarization in coronary slow flow:
Tp-e interval, Tpe/QT ratio, and Tp-e/QTc ratio. Ann Noninvasive Electrocardiol.

[8] Gupta P, Patel C, Patel H, Narayanaswamy S, Malhotra B, Green JT (2008). T(p-e)/QT ratio as an index of arrhythmogenesis. J Electrocardiol.

[9] Antzelevitch C, Viskin S, Shimizu W, Yan G-X, Kowey P, Zhang L (2007). Does Tpeak-Tend provide an index of transmural dispersion of
repolarization?. Heart Rhythm.

[10] Tatlisu MA, Özcan KS, Güngör B, Ekmekçi A, Çekirdekçi EI, Arugarslan E (2014). Can the T-peak to T-end interval be a predictor of mortality in
patients with ST elevation myocardial infarction. Coron Artery Dis.

[11] Castro Hevia J, Antzelevitch C, Tornés Bárzaga F, Dorantes Sánchez M, Dorticós Balea F, Zayas Molina R (2006). Tpeak-Tend and TpeakTend dispersion as risk factors for
ventricular tachycardia/ventricular fibrillation in patients with the
Brugada syndrome. J Am Coll Cardiol.

[12] Lang RM, Badano LP, Mor-Avi V, Afilalo J, Armstrong A, Ernande L (2015). Recommendations for cardiac chamber quantification by
echocardiography in adults: an update from the American Society of
Echocardiography and the European Association of Cardiovascular
Imaging. J Am Soc Echocardiogr.

[13] Simpson Jr RJ, Cascio WE, Schreiner PJ, Crow RS, Rautaharju PM, Heiss G (2002). Prevalence of premature ventricular contractions in a population
of African American and white men and women: The Atherosclerosis Risk In
Communities (ARIC) study. Am Heart J.

[14] Wang K, Hodges M (1992). The premature ventricular complex as a diagnostic
aid. Ann Intern Med.

[15] Shiraishi H, Ishibashi K, Urao N, Tsukamoto M, Hyogo M, Keira N (2002). A case of cardiomyopathy induced by premature ventricular
complexes. Circ J.

[16] Chugh SS, Shen WK, Luria DM, Smith HC (2000). First evidence of premature ventricular complex-induced
cardiomyopathy: a potentially reversible cause of heart
failure. J Cardiovasc Electrophysiol.

[17] Myerburg RJ, Kessler KM, Castellanos A (1993). Sudden cardiac death: epidemiology, transient risk, and
intervention assessment. Ann Intern Med.

[18] Tilz RR, Lin T, Makimoto H, Ouyang F (2014). Successful epicardial ablation of electrical storms due to
recurrent ventricular fibrillation triggered by premature ventricular
contractions. Heart Rhythm.

[19] Haïssaguerre M, Shah DC, Jaïs P, Shoda M, Kautzner J, Arentz T (2002). Role of Purkinje conducting system in triggering of idiopathic
ventricular fibrillation. Lancet.

[20] Tsuchiya T, Nakagawa S, Yanagita Y, Fukunaga T (2007). Transition from purkinje fiber-related rapid polymorphic
ventricular tachycardia to sustained monomorphic ventricular tachycardia in
a patient with a structurally normal heart: a case report. J Cardiovasc Electrophysiol.

[21] Nikolic G, Bishop RL, Singh JB (1982). Sudden death recorded during Holter monitoring. Circulation.

[22] Savelieva I, Wichterle D, Camm JA (2005). QT-interval turbulence induced by atrial and ventricular
extrastimuli in patients with ventricular tachycardia. Pacing Clin Electrophysiol.

[23] Adams JC, Srivathsan K, Shen WK (2012). Advances in management of premature ventricular
contractions. J Interv Card Electrophysiol.

[24] Lee GK, Klarich KW, Grogan M, Cha YM (2012). Premature ventricular contraction-induced cardiomyopathy: a
treatable condition. Circ Arrhythm Electrophysiol.

[25] Armaganijan LV, Staico R, Moreira DA, Lopes RD, Medeiros PT, Habib R (2015). 6-month outcomes in patients with implantable
cardioverter-defibrillators undergoing renal sympathetic denervation for the
treatment of refractory ventricular arrhythmias. JACC Cardiovasc Interv.

[26] DeBacker G, Jacobs D, Prineas R, Crow R, Vilandre J, Kennedy H (1979). Ventricular premature contractions: a randomized non-drug
intervention trial in normal men. Circulation.

[27] Dobmeyer DJ, Stine RA, Leier CV, Greenberg R, Schaal SF (1983). The arrhythmogenic effects of caffeine in human
beings. N Engl J Med.

[28] Sari I, Zengin S, Özer O, Davutoglu V, Yildirim C, Aksoy M (2008). Chronic carbon monoxide exposure increases electrocardiographic
P-wave and QT dispersion. Inhal Toxico.

[29] Shimizu H, Ohnishi Y, Inoue T, Yokoyama M (2001). QT and JT dispersion in patients with monomorphic or polymorphic
ventricular tachycardia/ ventricular fibrillation. J Electrocardiol.

[30] Soylu K, Inci S, Aksan G, Nar G, Yüksel EP, Ocal HS (2016). Evaluation of inhomogeneities of repolarization in patients with
psoriasis vulgaris. Arch Med Sci.

[31] Kaplan O, Kurtoglu E, Nar G, Yasar E, Gozubuyuk G, Dogan C (2015). Evaluation of Electrocardiographic T-peak to T-end Interval in
Subjects with Increased Epicardial Fat Tissue Thickness. Arq Bras Cardiol.

[32] Yamaguchi M, Shimizu M, Ino H, Terai H, Uchiyama K, Oe K (2003). T wave peak-to-end interval and QT dispersion in acquired long QT
syndrome: a new index for arrhythmogenicity. Clin Sci.

[33] Yayla Ç, Özcan F, Aras D, Turak O, Özeke Ö, Çay S (2017). Tp-e interval and Tp-e/QT ratio before and after catheter
ablation in patients with premature ventricular complexes. Biomark Med.

